# Impact of Prior Cancer on the Prognosis of Patients With Laryngeal Cancer: A Population-Based Study Using the Surveillance, Epidemiology, and End Results Database

**DOI:** 10.3389/fonc.2020.561330

**Published:** 2020-10-20

**Authors:** Kailin Chen, Lamei Tian, Yajun Li, Yi Jin, Huai Liu, Hui Wang

**Affiliations:** ^1^Key Laboratory of Translational Radiation Oncology, Department of Radiation Oncology, Hunan Cancer Hospital and The Affiliated Cancer Hospital of Xiangya School of Medicine, Central South University, Changsha, China; ^2^Department of Lymphoma and Hematology, Hunan Cancer Hospital and The Affiliated Cancer Hospital of Xiangya School of Medicine, Central South University, Changsha, China; ^3^Department of Radiation Oncology, Hunan Cancer Hospital and The Affiliated Cancer Hospital of Xiangya School of Medicine, Central South University, Changsha, China

**Keywords:** prior cancer, survival, SEER, outcome, laryngeal cancer

## Abstract

**Background:** Little is known about the clinical significance of laryngeal cancer as a subsequent tumor. We aimed to determine the impact of a prior cancer history on the prevalence and prognosis of patients with laryngeal cancer.

**Methods:** We retrospectively reviewed patients diagnosed with laryngeal cancer between 2004 and 2011 in the Surveillance, Epidemiology, and End Results (SEER) database. The *t*-test and chi-squared test were used to compare variables as appropriate. Matched 1:1 case control-adjusted Kaplan–Meier analyses and Cox regression models were performed to investigate the impact of prior cancer on overall survival (OS).

**Results:** Among 20,987 patients with laryngeal cancer, nearly one-fifth (*n* = 3,915, 18.65%) had a prior cancer. The top three common prior cancers were prostate (588, 28.1%), lung and bronchus (354, 16.9%), and head and neck (306, 14.6%). A total of 73.4% of the prior cancers were diagnosed within 5 years of the laryngeal cancer diagnosis. Compared to patients without prior cancer, a worse survival was significantly associated with a prior cancer among laryngeal cancer patients, regardless of the interval time of the prior cancer (log-rank tests *P* < 0.001). Furthermore, prior cancer was an independent predictor of worse OS based on the Cox regression model [hazard ratio (HR) = 1.396, 95% confidence interval, 1.336–1.458]. In addition, patients with prior lung and bronchus cancer tended to have the worst survival (log-rank tests *P* < 0.001).

**Conclusions:** Prior cancer has an adverse effect on clinical outcomes among patients with laryngeal cancer. These results suggest that individualized treatment should be seriously considered in patients with laryngeal cancer and a history of prior cancer, regardless of the interval time of prior cancer.

## Introduction

Laryngeal cancer is a significant clinical problem, with 177,422 estimated new cases and 94,771 estimated deaths worldwide in 2018 (1). The larynx has a complex regional anatomy and plays an important role in phonation, respiration, and swallowing. Therefore, achieving maximum disease control while preserving organ function is important for the treatment of laryngeal cancer, which means a unique set of therapeutic challenges. Care for laryngeal cancer patients requires a comprehensive evaluation and treatment plan in a multidisciplinary setting (2). For patients with limited disease, the cure rate can be as high as 80–90% (3). However, ~60% of patients are diagnosed with advanced disease (4). Concurrent chemoradiation has become the dominant treatment for advanced laryngeal cancer because of its higher rates of organ preservation compared to radiation therapy (RT) alone or induction chemotherapy followed by RT (5, 6). Despite the increasing use of chemotherapy and radiotherapy combinations in the majority of patients with advanced cancer, no improvement in overall survival has been found (5, 7, 8). Total laryngectomy is used to treat locally recurrent disease after chemoradiotherapy, especially for older or frail patients (6, 9).

Although participating in clinical trials is one of the treatment choices essential for management of these patients, according to recent estimates, the overall trial participation rate is only 8.1% (10). Clinical trial eligibility criteria are one of the most important barriers (11, 12). Prior cancer diagnosis as an exclusion criterion is common, which may result in a substantial proportion of otherwise eligible patients being excluded and thus limit enrollment (11, 13). It was reported that over 80% of lung cancer clinical trials initiated by the Eastern Cooperative Oncology Group (ECOG) excluded patients with prior cancer because of a long-held belief that a prior cancer diagnosis and treatment might interfere with study conduct or outcomes (11, 13). However, not all prior cancers have adverse effects on clinical outcomes (14, 15).

The number of cancer survivors is increasing rapidly as a result of the aging, population, expansion of cancer screening, and improvement in treatment of cancer (16). Twenty-five percent of older and over 10% of younger adults who was newly diagnosed with cancer have a prior cancer history (13). The prevalence of prior cancer ranges from 3.5 to 36.9% according to age and incident cancer type (13). As the number of cancer survivors increases, the impact of this exclusion criterion is more likely to grow. Therefore, understanding the prevalence of the prior cancer history among patients newly diagnosed with different types of cancer has important significance for cancer treatment and research.

However, no study has specifically assessed the prevalence of prior cancer among patients with laryngeal cancer and the impact of prior cancer on clinical outcomes. There is also little known about the clinical characteristics of laryngeal cancer patients with a prior cancer history. Therefore, we identified the prevalence and clinical characteristics of prior cancer among patients with laryngeal cancer and determined the impact factors of these patients on prognosis using the Surveillance, Epidemiology, and End Results (SEER) database.

## Methods

### Data Source

We used the SEER database, which covers ~9.4% of the U.S. population (https://seer.cancer.gov/, accession number: 15832-Nov2018). The SEER^*^Stat software version 8.3.6 was used to extract data from the database. The study population included patients diagnosed with laryngeal cancer from January 2004 to December 2011, as several employed covariates have been introduced in SEER since 2004 (17).

Patients, aged younger than 15 years at diagnosis, with only death certificate or autopsy records or without complete survival data and follow-up information, were excluded from the study. We extracted demographic, clinical, and pathological data from the SEER database, including age, gender, race, marital status, TNM stage, primary sites, pathology grade, and treatment. Race was divided into white, black, and others. We classified marital status as married, unmarried, or unknown. The TNM stage was based on the American Joint Committee on Cancer (AJCC, 6th edition) staging system.

### Measures

A history of prior cancer was determined from the SEER sequence number record, as described in a previous study (15). Briefly, a person's tumors are sequentially numbered within each SEER submission; the order is based on date of diagnosis and sequence. The interval time between laryngeal cancer and prior cancer was calculated by using the SEER survival months of the index cancer and the most recent of any prior cancers. The primary outcome was overall survival. We set December 31, 2016, as the follow-up cutoff date to ensure that all patients enrolled in the study were followed-up for at least 5 years.

### Statistical Analysis

We divided the patients into two groups based on prior cancer history. To reduce selection bias and confounding, a matched 1:1 case-control method was performed. Variables were matched based on age, gender, race, marital status, pathologic grade, primary site, AJCC stage (6th edition), and treatment. The characteristics were balanced after application of the matched 1:1 case control method. These case control pairs were also used in subsequent analyses.

Variables were examined for association with or without prior cancer history by univariate analysis with the Pearson chi-squared analysis or the *t*-test. The multivariate logistic regression analysis was performed to identify whether prior cancer independently impacted prognosis. Survival curves were calculated by the Kaplan–Meier method and compared with the log-rank test. The prognostic importance of factors was analyzed using the Cox regression model (18). An enter procedure was used for multivariate analysis. Statistical significance was defined as *P* < 0.05 (two tailed). Statistical analyses were performed with IBM SPSS Statistics version 23.0 software (IBM Co, Armonk, New York, USA).

## Results

In total, we identified 20,987 eligible laryngeal cancer patients diagnosed between 2004 and 2011. Among these, 18.65% (*n* = 3,915) had a prior cancer history. Compared with patients without prior cancer, patients with prior cancer tended to be older (71 vs. 63 years, *P* < 0.001), female (22.0% vs. 19.1%, *P* < 0.001), white (84.2 vs. 81.1%, *P* < 0.001), and married (55.7 vs. 53.4%, *P* = 0.003). Patients with prior cancer also had a higher rate of being in the early stages of the disease at diagnosis. In both groups, the common sites of laryngeal cancer at diagnosis were the glottis and supraglottis. Moderately differentiated was the most common pathological grade. The vast majority of patients received surgical surgery. However, the percentage of radiotherapy was larger among patients with a history of prior cancer (30.1 vs. 20.6%, *P* < 0.001); patients with prior cancer also received more chemotherapy (76.1 vs. 65.8%, *P* < 0.001). The baseline characteristics are shown in Table 1. The characteristics were well balanced between the patients with or without prior cancer when using the case-control matching method (Table 1, matched-1 data set).

**Table 1:** Baseline characteristics of patients with larynx cancer.

**Characteristics**	**Original data set**			**Matched-1 data set**			**Matched-2 data set**		
	**No prior cancer**	**With prior cancer**	***P*-value**	**No prior cancer**	**With prior cancer**	***P*-value**	**No prior cancer**	**With prior cancer**	***P*-value**
	***N* = 17,072 (%)**	***N* = 3,915 (%)**		***N* = 2,745 (%)**	***N* = 2,745 (%)**		***N* = 1,498 (%)**	***N* = 1,498 (%)**	
Age at diagnosis (median)	63 (15.103)	71 (27.99)		70 (39.95)	70 (37.96)		69 (39.93)	70 (37.94)	
Age group, *N* (%)			<0.001			0.139			0.131
<65	9,517 (55.7)	1,105 (28.2)		836 (30.5)	786 (28.6)		475 (31.7)	437 (29.2)	
≥65	7,555 (44.3)	2,810 (71.8)		1,909 (69.5)	1,959 (71.4)		1,023 (68.3)	1,061 (70.8)	
Gender, *N* (%)			<0.001						
Female	3,257 (19.1)	860 (22.0)		461 (16.8)	461 (16.8)	1	214 (14.3)	214 (14.3)	1
Male	13,815 (80.9)	3,055 (78.0)		2,284 (83.2)	2,284 (83.2)		1,284 (85.7)	1,284 (85.7)	
Race, *N* (%)									
White	13,850 (81.1)	3,297 (84.2)	<0.001	2,458 (89.5)	2,458 (89.5)	1	1,317 (87.9)	1,317 (87.9)	1
Black	2,536 (14.9)	508 (13.0)		253 (9.2)	253 (9.2)		161 (10.7)	161 (10.7)	
Other	615 (3.6)	109 (2.8)		34 (1.2)	34 (1.2)		20 (1.3)	20 (1.3)	
Unknown	71 (0.4)	1 (0.0)		NA	NA		NA	NA	
Marital status, *N* (%)			0.003			1			1
Married	9,124 (53.4)	2,179 (55.7)		1,631 (59.4)	1,631 (59.4)		887 (59.2)	887 (59.2)	
Unmarried	7,152 (41.9)	1,530 (39.1)		1,036 (37.7)	1,036 (37.7)		567 (37.9)	567 (37.9)	
Unknown	796 (4.7)	206 (5.3)		78 (2.8)	78 (2.8)		44 (2.9)	44 (2.9)	
AJCC TNM			<0.001			1			1
I	6,060 (35.5)	1,690 (43.2)		1,383 (50.4)	1,383 (50.4)		743 (49.6)	743 (49.6)	
II	2,707 (15.9)	600 (15.3)		403 (14.7)	403 (14.7)		229 (15.3)	229 (15.3)	
III	2,644 (15.5)	567 (14.5)		354 (12.9)	354 (12.9)		200 (13.4)	200 (13.4)	
IV	4,316 (25.3)	704 (18.0)		452 (16.5)	452 (16.5)		255 (17.0)	255 (17.0)	
Unknown	1,345 (7.9)	354 (9.0)		153 (5.6)	153 (5.6)		71 (4.7)	71 (4.7)	
Primary site, *N* (%)			<0.001			1			1
Glottis	9,226 (54.0)	2,147 (54.8)		1,749 (63.7)	1,749 (63.7)		902 (60.2)	902 (60.2)	
Supraglottis	5,893 (34.5)	1,329 (33.9)		887 (32.3)	887 (32.3)		533 (35.60	533 (35.60	
Subglottis	286 (1.7)	95 (2.4)		13 (0.5)	13 (0.5)		9 (0.6)	9 (0.6)	
Laryngeal cartilage	115 (0.7)	40 (1.0)		1 (0.0)	1 (0.0)		NA	NA	
Overlapping lesion of larynx	427 (2.5)	82 (2.1)		18 (0.7)	18 (0.7)		10 (0.7)	10 (0.7)	
Larynx, NOS	1,125 (6.6)	222 (5.7)		77 (2.8)	77 (2.8)		44 (2.9)	44 (2.9)	
Grade, *N* (%)			0.209			1			1
Well differentiated; Grade I	2,394 (14.0)	504 (12.9)		346 (12.6)	346 (12.6)		175 (11.7)	175 (11.7)	
Moderately differentiated; Grade II	7,848 (46.0)	1,798 (45.9)		1,375 (50.1)	1,375 (50.1)		749 (50.0)	749 (50.0)	
Poorly differentiated; Grade III	2,941 (17.2)	669 (17.1)		403 (14.7)	403 (14.7)		229 (15.3)	229 (15.3)	
Undifferentiated; anaplastic; Grade IV	118 (0.7)	32 (0.8)		5 (0.2)	5 (0.2)		3 (0.2)	3 (0.2)	
Unknown	3,771 (22.1)	912 (23.3)		616 (22.4)	616 (22.4)		342 (22.8)	342 (22.8)	
Surgery of primary site, *N* (%)			0.275			1			1
No/unknown	16,983 (99.5)	3,889 (99.3)		2,744 (100)	2,744 (100)		1,498 (100)	1,498 (100)	
Yes	89 (0.5)	26 (0.7)		1 (0.0)	1 (0.0)		NA	NA	
Radiation, *N* (%)			<0.001			1			
No/unknown	3,516 (20.6)	1,180 (30.1)		628 (22.9)	628 (22.9)		339 (22.6)	339 (22.6)	
Yes	13,556 (79.4)	2,735 (69.9)		2,117 (77.1)	2,117 (77.1)		1,159 (77.4)	1,159 (77.4)	
Chemotherapy, *N* (%)			<0.001			1			1
No/unknown	11,225 (65.8)	2,979 (76.1)		2,130 (77.6)	2,130 (77.6)		1,141 (76.2)	1,141 (76.2)	
Yes	5,847 (34.2)	936 (23.9)		615 (22.4)	615 (22.4)		357 (23.8)	357 (23.8)	

*NA, not available*.

**Figure 1:**
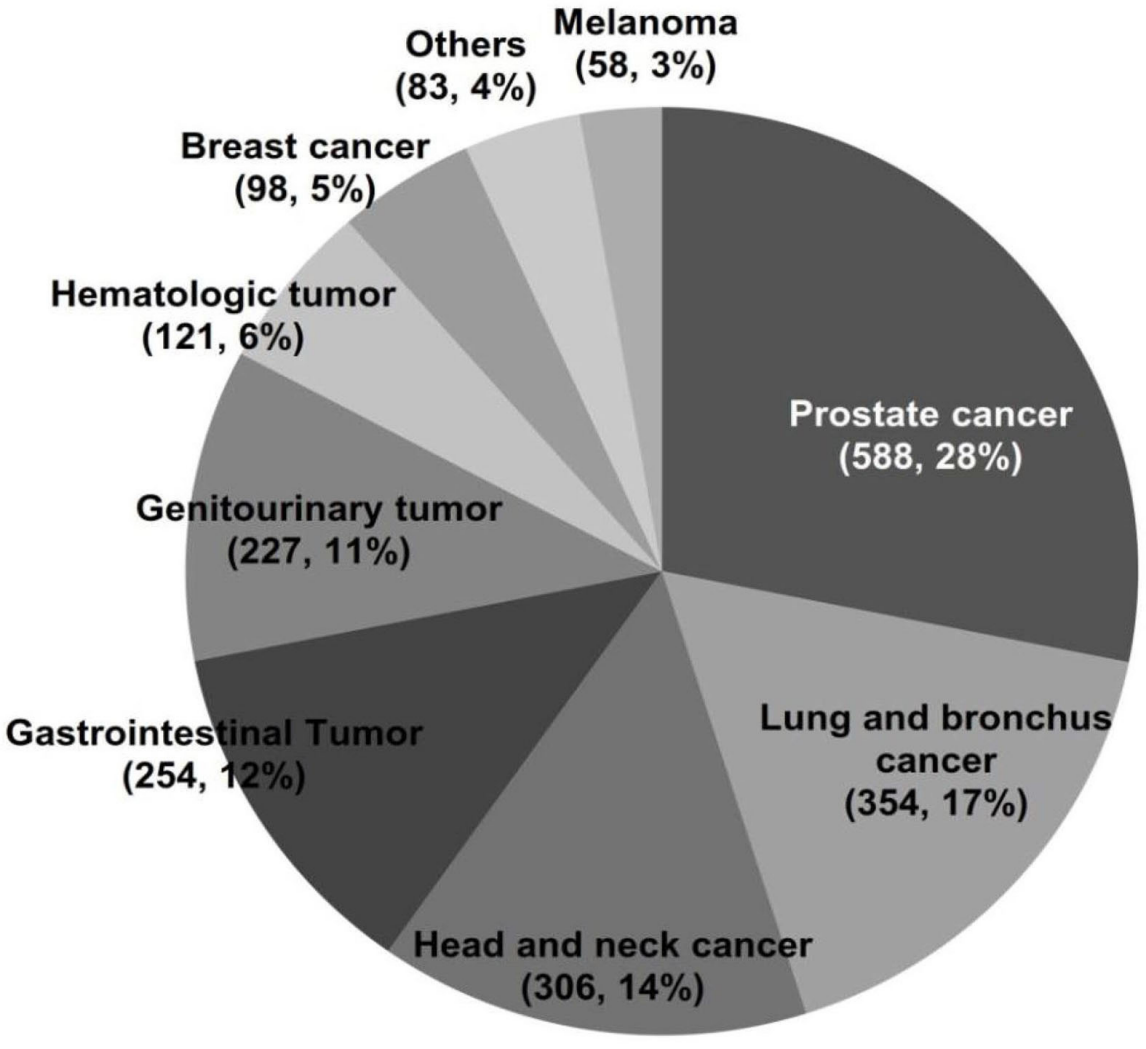
Distribution of prior cancers in 2,089 patients with laryngeal cancer.

Among 3,915 laryngeal cancer patients with a cancer history, the types of prior cancer were clearly recorded for 2,098 patients in the SEER database. The types of previous tumors are shown in Figure 1. Over 73.4% of the prior cancers were diagnosed within 5 years of the index laryngeal cancer. The median time between the most recent prior cancer diagnosis and the index laryngeal cancer was 32 months. The case-control matching method was also applied between patients without prior cancer and patients with prior cancer and specific site records (Table 1, matched-2 data set). Patients with prior cancer who had shorter interval times were older and tended to have a primary site of the supraglottis compared with patients with longer interval times. Lung and bronchus cancer was common in patients with shorter interval times. The detailed information is shown in Table 2.

**Table 2:** Characteristics of larynx cancer patients with prior cancer.

**Characteristics**	**Patients with prior cancer in matched data set 2**		
	**Interval time <60 months**	**Interval time ≥ 60 months**	***P*-value**
	***N* = 1,100 (%)**	***N* = 398 (%)**	
Age group			<0.001
<65	351 (31.9)	86 (21.6)	
≥65	749 (68.1)	312 (78.4)	
Gender			0.866
Female	158 (14.4)	56 (14.1)	
Male	942 (85.6)	342 (85.9)	
Race			
White	965 (87.7)	352 (88.4)	0.826
Black	121 (11.0)	40 (10.1)	
Other	14 (1.3)	6 (1.5)	
Unknown	NA	NA	
Marital status			0.079
Married	634 (57.6)	253 (63.6)	
Unmarried	435 (39.5)	132 (33.2)	
Unknown	31 (2.8)	13 (3.3)	
AJCC TNM			0.605
I	537 (48.8)	206 (51.8)	
II	166 (15.1)	63 (15.8)	
III	150 (13.6)	50 (12.6)	
IV	190 (17.3)	65 (16.3)	
Unknown	57 (5.2)	14 (3.5)	
Primary site			0.003
Glottis	631 (57.4)	271 (68.1)	
Supraglottis	422 (38.4)	111 (27.9)	
Subglottis	8 (0.7)	1 (0.3)	
Laryngeal cartilage	NA	NA	
Overlapping lesion of larynx	8 (0.7)	2 (0.5)	
Larynx, NOS	31 (2.8)	13 (3.3)	
Grade			0.46
Well differentiated; Grade I	126 (11.5)	49 (12.3)	
Moderately differentiated; Grade II	540 (49.1)	209 (52.5)	
Poorly differentiated; Grade III	179 (16.3)	50 (12.6)	
Undifferentiated; anaplastic; Grade IV	2 (0.2)	1 (0.3)	
Unknown	253 (23.0)	89 (22.4)	
Surgery of primary site			NA
No/unknown	1,100 (100)	398 (100)	
Yes	NA	NA	
Radiation			0.57
No/unknown	253 (23.0)	86 (21.6)	
Yes	847 (77.0)	312 (78.4)	
Chemotherapy			0.281
No/unknown	830 (75.5)	311 (78.1)	
Yes	270 (24.5)	87 (21.9)	
Sties of prior cancer			<0.001
Breast	41 (3.7)	20 (5.0)	
Gastrointestinal tumor	135 (12.3)	53 (13.3)	
Genitourinary tumor	124 (11.3)	54 (13.6)	
Head and neck cancer	126 (11.5)	46 (11.6)	
Hematologic tumor	67 (6.1)	19 (4.8)	
Lung and bronchus cancer	210 (19.1)	29 (7.3)	
Melanoma	29 (2.6)	15 (3.8)	
Prostate cancer	320 (29.1)	155 (38.9)	
Other	48 (4.4)	7 (1.8)	

*NA, not available*.

**Figure 2:**
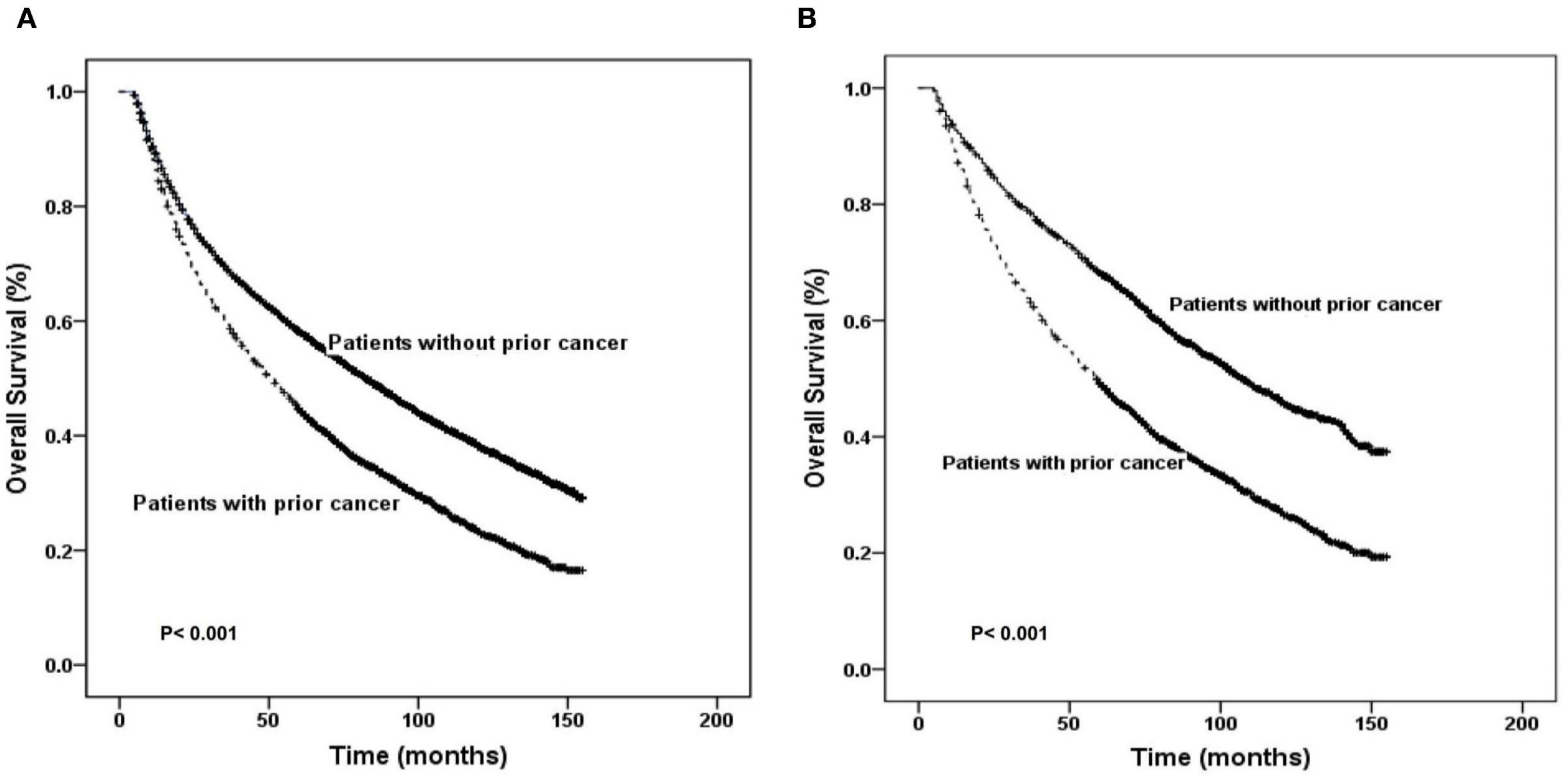
Kaplan–Meier survival curves of prior cancer impact on overall survival in patients with laryngeal cancer. Prior cancer was significantly associated with worse survival in laryngeal patients with compared to no prior cancer in both unadjusted and adjusted Kaplan–Meier analyses (**A,B**, respectively).

In an unadjusted Kaplan–Meier analysis, compared to no prior cancer, prior cancer was significantly associated with worse survival among laryngeal cancer patients (log-rank tests *P* < 0.001) (Figure 2A). The overall 5-year survival rates for patients with or without prior cancer were 44.7% [95% confidence interval (CI), 43.1–46.3] and 58.2% (95% CI, 57.4–60.0), respectively. In an adjusted Kaplan–Meier analysis, patients with a history of prior cancer also had inferior OS than patients without a history of prior cancer (log-rank tests *P* < 0.001) (Figure 2B).

The overall 5-year survival rates for cancer of the glottis, supraglottis, subglottis, laryngeal cartilage, overlapping lesion of larynx, and larynx, NOS were 65.6% (95% CI, 64.8–66.4), 44.0% (95% CI, 42.8–45.2), 48.8% (95% CI, 43.7–53.9), 63.2% (95% CI, 55.6–70.7), 41.6% (95% CI, 37.3–45.9), and 40.5% (95% CI, 38.0–43.0), respectively. Carcinoma of the glottis patients tended to have the best survival (log-rank tests *P* < 0.001). Subgroup analyses for patients with different tumor location indicated that prior cancer has an adverse effect on clinical outcomes among laryngeal cancer patients except the cancer occurred in the overlapping lesion of larynx.

**Figure 3:**
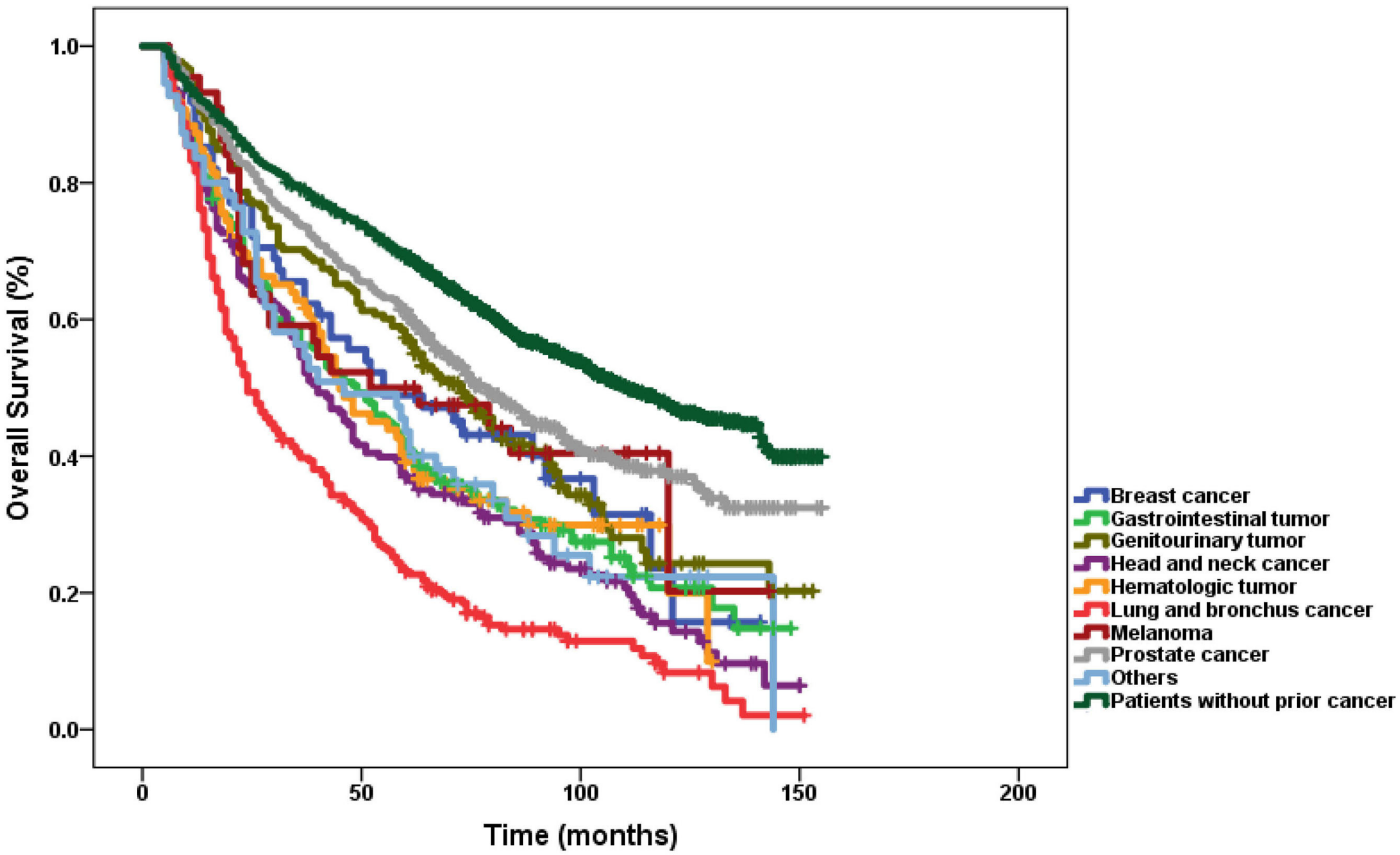
Subgroup analysis of the impact of prior cancer on overall survival stratified by the prior cancer type in laryngeal cancer.

Figure 3 displays OS according to different types of prior cancer based on adjusted Kaplan–Meier analysis of the matched-2 data set. Among those with a history of prior cancer, patients with prior lung and bronchus cancer tended to have the worst survival (log-rank tests *P* < 0.001). Using the log rank method to compare the survival of different groups, only patients with prior melanoma seemed to have no significant difference in survival compared with patients without prior cancer.

**Figure 4:**
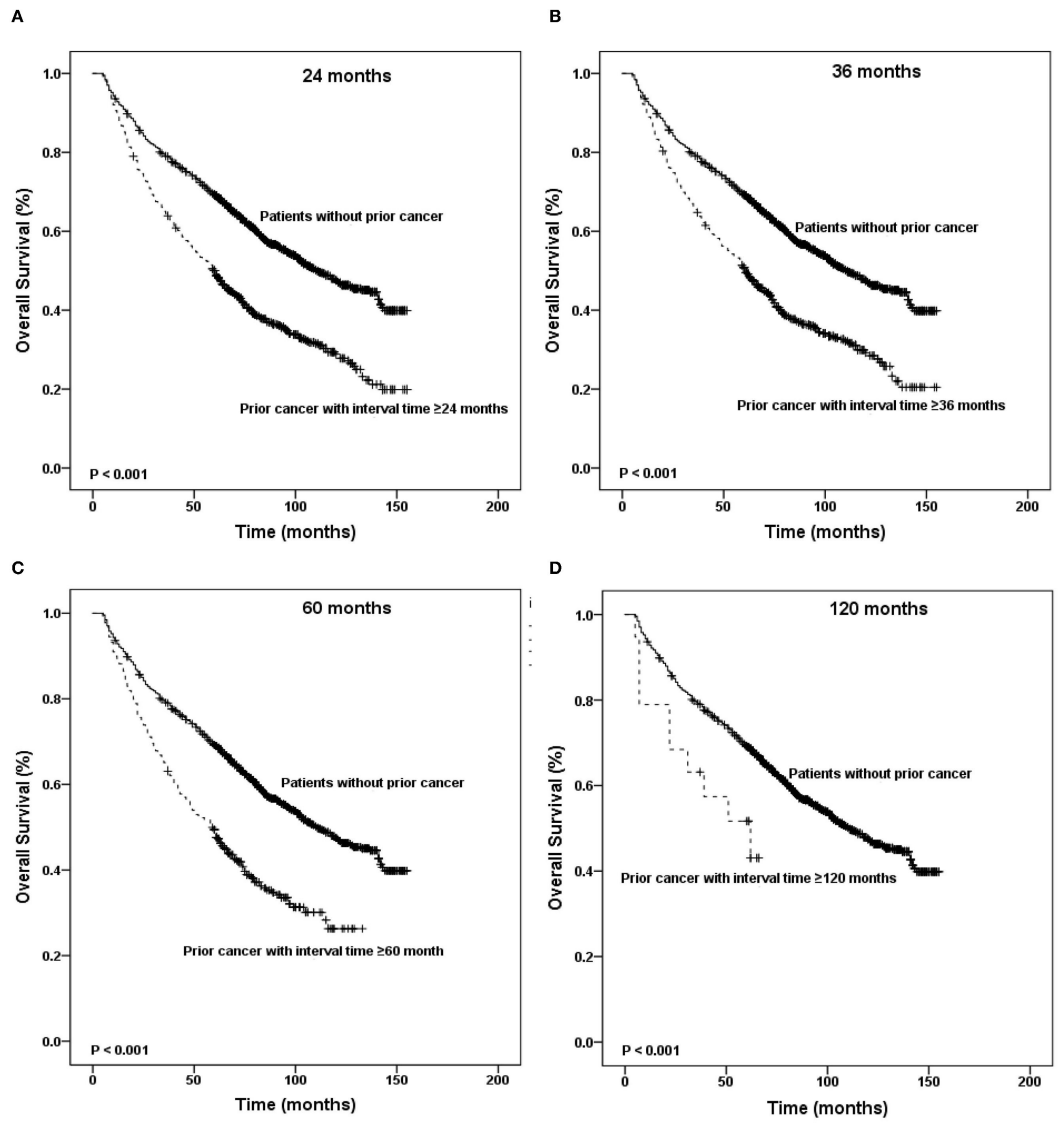
Kaplan-Meier survival curves between patients without prior cancer and patients with different timing of prior cancer. Patients with prior cancer had a worse survival to patients without prior cancer, regardless of the interval timing of the prior cancer.

**Figure 5:**
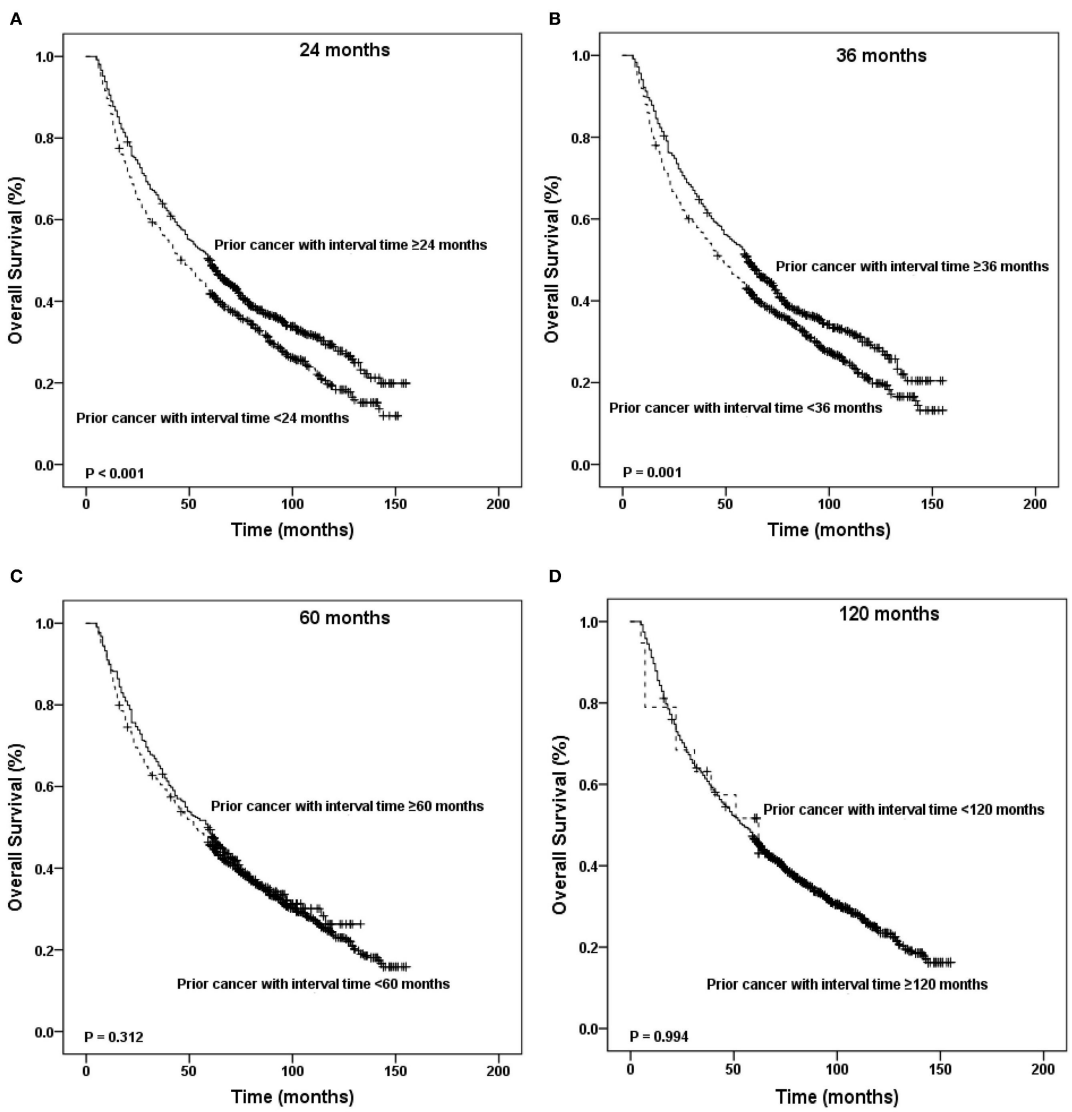
Kaplan-Meier survival curves according to the different timing of prior cancer. Patients with interval times longer than 2, 3, 5, or 10 years had worse survival than patients without prior cancer.

Figures 4, 5 show the Kaplan–Meier survival curves stratified by the timing of prior cancer in the matched-2 data set. Laryngeal cancer patients with a history of prior cancer show inferior survival compared with patients with no prior cancer. Taking the timing of prior cancer into consideration, the subgroup analysis showed that patients with prior cancer had a worse survival to patients without prior cancer, regardless of the interval timing of the prior cancer. Patients with interval times longer than 2, 3, 5, or 10 years had worse survival than patients without prior cancer (*P* < 0.001) (Figure 4).

In addition, patients with interval times shorter than 2 or 3 years had worse survival than patients with interval times longer than 2 or 3 years (*P* < 0.001). Patients with interval times shorter than 5 or 10 years had the same survival that patients with interval times longer than 5 or 10 years did (*P* = 0.312 and *P* = 0.994, respectively) (Figure 5).

In unadjusted Cox models, patients with a history of prior cancer had a poorer OS [hazard ratio (HR) = 1.396, 95% confidence interval, 1.336–1.458] than patients with no prior cancer (Table 3). The same tendency was found in matched-1 Cox models.

**Table 3:** Cox regression analysis of the impact of prior cancer history on patients with larynx cancer.

**Characteristics**	**Original data set**			**Matched data set**		
	**Univariate analysis**	**Multivariate analysis**		**Univariate analysis**	**Multivariate analysis**	
	***P*-value**	**Hazard ratio (95% CI)**	***P*-value**		**Hazard ratio (95% CI)**	***P*-value**
Age group	<0.001			<0.001		
<65		Reference			Reference	
≥65		1.783 (1.718, 1.851)	<0.001		1.955 (1.793, 2.131)	<0.001
Gender	0.069			0.21		
Female		Reference			Reference	
Male		1.174 (1.122, 1.229)	<0.001		1.146 (1.037, 1.265)	0.007
Race	<0.001			0.138		
White		Reference			Reference	
Black		2.952 (1.747, 4.989)	<0.001		1.039 (0.724, 1.493)	0.834
Other		3.104 (1.834, 5.255)	<0.001		1.057 (0.724, 1.542)	0.775
Unknown		2.709 (1.588, 4.622)	<0.001		NA	NA
Marital status	<0.001			<0.001		
Married		Reference			Reference	
Unmarried		0.896 (0.820, 0.980)	0.016		0.869 (0.696, 1.084)	0.214
Unknown		1.218 (1.113, 1.332)	<0.001		1.199 (0.957, 1.503)	0.114
AJCC TNM	<0.001			<0.001		
I		Reference			Reference	
II		0.789 (0.734, 0.849)	<0.001		0.765 (0.654, 0.896)	0.001
III		1.014 (0.937, 1.096)	0.733		0.997 (0.837, 1.188)	0.973
IV		1.255 (1.158, 1.360)	<0.001		1.269 (1.057, 1.523)	0.011
Unknown		1.716 (1.589, 1.854)	<0.000		1.636 (1.367, 1.958)	<0.001
Primary Site	<0.001			<0.001		
Glottis		Reference			Reference	
Supraglottis		0.647 (0.602, 0.696)	<0.001		0.586 (0.479, 0.717)	<0.001
Subglottis		0.935 (0.871, 1.004)	0.062		0.848 (0.693, 1.038)	0.111
Laryngeal cartilage		0.817 (0.711, 0.938)	0.004		0.569 (0.335, 0.965)	0.036
Overlapping lesion of larynx		0.602 (0.474, 0.764)	<0.001		0.001 (0, 0.000842)	0.805
Larynx, NOS		0.894 (0.792, 1.009)	0.069		0.958 (0.629, 1.458)	0.841
Grade	<0.001			<0.001		
Well differentiated; Grade I		Reference			Reference	
Moderately differentiated; Grade II		0.883 (0.828, 0.942)	<0.001		0.951 (0.834, 1.084)	0.449
Poorly differentiated; Grade III		0.988 (0.943, 1.035)	0.615		1.015 (0.925, 1.113)	0.760
Undifferentiated; anaplastic; Grade IV		1.106 (1.045, 1.170)	<0.001		1.098 (0.973, 1.240)	0.130
Unknown		1.347 (1.116, 1.627)	0.002		1.509 (0.675, 3.376)	0.317
Surgery of Primary Site	0.004			0.669		
No/unknown		Reference			Reference	
Yes		1.394 (1.122, 1.730)	0.003		2.716 (0.675, 10.929)	0.160
Radiation	0.18			0.132		
No/unknown		Reference			Reference	
Yes		0.894 (0.859, 0.931)	<0.001		1.021 (0.933, 1.118)	0.647
Chemotherapy	<0.001			<0.001		
No/unknown		Reference			Reference	
Yes		1.061 (1.014, 1.110)	0.010		0.839 (0.751, 0.937)	0.002
Prior cancer	<0.001			<0.001		
No		Reference			Reference	
Yes		1.396 (1.336, 1.458)	<0.001		1.794 (1.670, 1.927)	<0.001

*NA, not available*.

## Discussion

Randomized controlled trials (RCTs) are often considered to be the gold standard for evaluation of different interventions for oncology. The beneficial effects of some interventions are largely determined by factors such as patient characteristics (19). In fact, little is known about how much a history of prior cancer and its treatment impact cancer outcomes, partly because patients who have a prior cancer history are usually excluded from clinical trials and partly because of the potential interference of study outcomes (11). Murphy et al. described the prevalence of prior cancer among persons newly diagnosed with cancer (13). Zhou et al. found that OS among patients, with cancers such as colon and rectum, bone and soft tissue, breast cancers, and melanoma, who had a prior cancer history was inferior to the OS of patients without a prior cancer. Patients with cancer of the nasopharynx, esophagus, stomach, gallbladder, liver, and lung with a history of prior cancer showed similar OS to that of patients with no prior cancer (15). These authors further confirmed that prior cancer does not affect the outcome among patients with nasopharyngeal carcinoma (20). However, there are no data currently available to determine whether laryngeal cancer patients with prior cancer face a worse prognosis. Therefore, we evaluated the prevalence and the impact of a prior cancer diagnosis on the prognosis among patients with laryngeal cancer.

We observed that laryngeal cancer patients with prior cancer had poorer survival outcomes than those without prior cancer. Multivariate analysis showed that a history of prior cancer was an independent prognostic factor. To our knowledge, this is the first study to evaluate the characteristics and the impact of prior cancer on the prognosis among laryngeal cancer patients.

In our study, nearly one-fifth (18.65%) had a prior cancer, which is relatively high according to previously reported incidences of prior cancer of other tumor types (13, 14). Prostate cancer and lung and bronchus cancer were common prior cancers, accounting for 28.1 and 16.9% of all prior cancers, respectively. These cancer types are complex diseases and may share many risk factors, such as aging, smoking, and alcohol consumption. Indeed, alcohol consumption is one of the most important risk factors for human cancers (21, 22). Smoking is also an established risk factor for lung and bronchus cancer and laryngeal cancer (23); it may also elevate levels of circulating androsterone and testosterone, which may increase prostate cancer risk and contribute to cancer progression (24). HPV infection also contributes to the cancer of the larynx (25). The underlying molecular and biological characteristics may differ among laryngeal cancer patients with or without prior lung cancer or other types of cancers. Moreover, the 5-year OS rate of primary lung cancer is significantly worse than that of prostate cancer according to the results of the latest epidemiological research, which is 19 vs. 98%, respectively (26). A large part of laryngeal cancer patients with prior lung cancer may eventually die due to the recurrence and progression of lung cancer, while prior prostate cancer has little effect on the survival of patients with laryngeal cancer. These may lead to the different survival of laryngeal cancer only and laryngeal cancer patients with other prior cancers, which may partly explain the influence of different prior cancers on the prognosis of patients with laryngeal cancer.

Prior cancer was significantly associated with worse survival among laryngeal cancer patients compared to patients without prior cancer in both unadjusted and adjusted Kaplan–Meier analyses. A previous study also confirmed that a prior cancer history might impact OS in patients with prostate cancer (15). In this cohort, less treatment was associated with subsequent prostate cancer than primary prostate cancer (27). Ji et al. also found that overall survival rates of breast cancer were significantly lower in women with breast cancer as the second primary cancer than in those with breast cancer as the prior cancer (28). Several studies have mentioned that prior cancer shows a lack of adverse effects on survival outcomes in many other cancer types, such as nasopharyngeal carcinoma, esophageal cancer, gastrointestinal tract, lung, glioblastoma, and pancreatic cancers (14, 15, 29–34). In addition, over 70% of prior cancers occurred within 5 years of the index laryngeal cancer in our study. The median interval between prior cancer and the index laryngeal cancer was 32 months. The subgroup analysis found that laryngeal cancer patients with prior cancer had inferior survival compared with patients without a prior cancer history, regardless of the interval time of the prior cancer. Moreover, among patients with different types of prior cancer, those with prior lung and bronchus cancer had the worst OS. Therefore, laryngeal cancer patients with a prior cancer history should be carefully considered for enrollment of clinical trials.

There are, however, some limitations to the present study. First, selection bias is inevitable in a retrospective study. Although we performed a matched 1:1 case control method to minimize such bias, residual confounding factors could not be entirely ruled out because of hidden biases. Second, we could not obtain detailed data on treatment-related factors of prior cancer and comorbidities from the SEER database.

In conclusion, prior cancer has an adverse effect on clinical outcomes among patients with laryngeal cancer, regardless of the interval time of the prior cancer. These results suggest that individualized treatment should be seriously considered in laryngeal cancer patients with a history of prior cancer, regardless of the interval time of the prior cancer. Further studies with more data are warranted to confirm our findings.

## Data Availability Statement

The datasets presented in this study can be found in online repositories. The names of the repository/repositories and accession number(s) can be found in the article/supplementary material.

## Author Contributions

KC, HW, and YL: design. KC, YJ, HL, and LT: data analysis. KC and YL wrote the manuscript. All authors contributed to the article and approved the submitted version.

## Conflict of Interest

The authors declare that the research was conducted in the absence of any commercial or financial relationships that could be construed as a potential conflict of interest.
